# Effects of levosimendan on cellular metabolic alterations in patients with septic shock: a randomised controlled study

**DOI:** 10.1186/2197-425X-3-S1-A811

**Published:** 2015-10-01

**Authors:** B Meddeb, Z Hajjej, C Romdhani, I Labbene, M Ferjani

**Affiliations:** Military Hospital of Tunis, Tunis, Tunisia

## Introduction

Mitochondrial dysfunction and consequent cellular energetic failure play a key role in the development of sepsis-related organs failure. Levosimendan, by a calcium-sensitizing mechanism increases myocardial contractility while simultaneously exerting vasodilatory properties via activation of ATP-dependent potassium channels (KATP) [1].

## Objectives

The aim of the present study was to evaluate the effects of levosimendan on muscle metabolism compared with placebo and dobutamine in patients with spetic shock.

## Methods

The study was designed as a prospective, double-blind, placebo-controlled, clinical trial and performed in a Tunisian medical surgical intensive care unit. After achieving normovolemia and a mean arterial pressure of at least 65 mmHg, 30 septic shock patients were randomized to one of three intravenous treatment groups: levosimendan 0.2 µg/kg/min (n = 10), dobutamine 5 µg/kg/min (n = 10) or placebo (n = 10). Systemic hemodynamic monitoring of the patients included a central venous catheter, a radial artery catheter and a pulmonary artery catheter. A microdialysis probe was placed into the femoral quadriceps and samples were collected at baseline (before drugs adminstration) and every 6 hours for 3 days. The changes in the energy-related metabolites lactate, the lactate/pyruvate ratio, glucose and glycerol were analyzed. concomitantly with dialysate sampling, the effects on global haemodynamics, were assessed. Lactate and L/P clearances were also calculated.

## Results

Baseline characteristics, including age, gender, body weight, and Cause of septic shock, as well as onset time of septic shock, IGSII, and mortality were not different among groups. In addition, there was no significant difference between groups at baseline (H0) in any of the investigated hemodynamic or MD metabolites variables. Levosimendan group had a greater decrease in L/P ratio and a greater increase in MD pyruvate at the 72 th 72th hour compared with the placebo group (p = 0.043). Tissue lactate clearance was significantly increased in the levosimendan arm compared with the placebo arm at 54th, 60th and 72th hours of the study ( H_54_ p = 0.036 ; H_60_ p = 0.043 ; H_72_ p = 0.041). From 48th to 72th hours, Tissue L/P clearance was significantly higher in levosimendan-treated patients compared with placebo (H_48_ (p = 0.015), H_54_ (p = 0.032), H_60_ (p = 0.016) et à H_72_ (p = 0.007)). Tissue L/P clearance Tissue L/P clearance was higher in Levosimendan group than in dobutamine group at 72th hour (p = 0.006).

## Conclusions

Our results demonstrate that levosimendan improves Cellular metabolic alterations.

Our data suggest that levosimendan may protect mitochondria from oxidative stress and maintain cellular energy homeostasis during septic shock by opening mitochondrial KATP channels.
